# Old Challenges or New Issues? Genetic Health Professionals’ Experiences Obtaining Informed Consent in Diagnostic Genomic Sequencing

**DOI:** 10.1080/23294515.2020.1823906

**Published:** 2020-10-05

**Authors:** Danya F. Vears, Pascal Borry, Julian Savulescu, Julian J. Koplin

**Affiliations:** a Melbourne Law School, University of Melbourne, Parkville, Australia; b Biomedical Ethics Research Group, Murdoch Children’s Research Institute, Parkville, Australia; c Department of Public Health and Primary Care, Center for Biomedical Ethics and Law, Leuven, Belgium; d Leuven Institute for Human Genetics and Society, Leuven, Belgium; e Oxford Uehiro Centre for Practical Ethics, University of Oxford, Oxford, UK; f Wellcome Centre for Ethics and Humanities, University of Oxford, Oxford, UK

**Keywords:** Genetic research, medicine, human subjects research, clinical genetics, informed consent

## Abstract

**Background:**

While integrating genomic sequencing into clinical care carries clear medical benefits, it also raises difficult ethical questions. Compared to traditional sequencing technologies, genomic sequencing and analysis is more likely to identify unsolicited findings (UF) and variants that cannot be classified as benign or disease-causing (variants of uncertain significance; VUS). UF and VUS pose new challenges for genetic health professionals (GHPs) who are obtaining informed consent for genomic sequencing from patients.

**Methods:**

We conducted semi-structured interviews with 31 GHPs across Europe, Australia and Canada to identify some of these challenges.

**Results:**

Our results show that GHPs find it difficult to prepare patients to receive results because a vast amount of information is required to fully inform patients about VUS and UF. GHPs also struggle to engage patients – many of whom may be focused on ending their ‘diagnostic odyssey’ – in the informed consent process in a meaningful way. Thus, some questioned how ‘informed’ patients actually are when they agree to undergo clinical genomic sequencing.

**Conclusions:**

These findings suggest a tension remains between sufficient information provision at the risk of overwhelming the patient and imparting less information at the risk of uninformed decision-making. We suggest that a shift away from ‘fully informed consent’ toward an approach aimed at realizing, as far as possible, the underlying goals that informed consent is meant to promote.

## Introduction

Genomic sequencing (which includes genome sequencing, exome sequencing, and gene sequencing panels) has revolutionized the identification of genetic causes of disease in research and is now well embedded in the clinical setting, particularly in areas such as rare disease and cancer (Prokop et al. 2018). Genomic sequencing carries many benefits for clinical care. It increases the rate of identification of disease-causing variants in patients with genetic conditions, which can lead to more accurate diagnosis, better patient management or treatment, and a more informative prognosis (Stark et al. 2018). Use of genomic sequencing can result in identification of the genetic cause in up to 57.5% of patients, depending on the condition and selection criteria used (Yang et al. 2013; Lee et al. 2014; Soden, Saunders et al. 2014; Yang, Muzny et al. 2014; Daoud et al. 2016; Stark, Tan et al. 2016). This success rate has been shown to decrease the time to diagnosis, which leads to other benefits including a reduction in unnecessary (and often invasive) tests and an improvement in the quality of care (Stark et al. 2018).

Yet, the introduction of genomic sequencing has also added layers of complexity to the field of genomic medicine. The nature of the testing, both in its ability to sequence many genes at the same time and the speed at which the sequence of each patient can be obtained, means that data analysis is operating at a much larger scale. There is also a much greater likelihood of identifying variants that cannot be classified as either benign or pathogenic, referred to as variants of uncertain significance or VUS, increasing the uncertainty in relation to the results that may be returned (Turbitt et al. 2018; Vears et al. 2018). Genomic sequencing has also led to an increased chance of inadvertently identifying variants in disease-causing genes unrelated to the clinical question, referred to as unsolicited findings (UF). This is particularly the case with exome and genome sequencing, although UF can still be identified when using more targeted sequencing approaches. Identification of UF for some conditions can lead to early detection via surveillance, access to early treatments, or other strategies to prevent disease onset. Research shows wide variation in whether laboratories report such findings (Hehir-Kwa et al. 2015; Vears, Sénécal et al. 2017), which has implications for equity in patients’ ability to access potentially life-saving interventions. Some have also questioned whether patients are in fact capable of making free choices about receiving UF when they are offered the option (Viberg et al. 2016).

In addition, genomic sequencing also raises the possibility of actively searching for a specific list of disease-causing variants where the evidence of benefit (via surveillance or prevention) from early detection is considered sufficient to warrant testing. When they are actively sought, such variants are referred to as secondary findings (SF) (Green et al. 2013; Turbitt et al. 2018; Vears et al. 2018). Although many laboratories in the United States offer to test for SF routinely when a request for diagnostic genomic sequencing is made, this is much less common in other parts of the world, such as Europe, Australia, and Canada (Vears, Sénécal et al. 2017). The concept of searching for SF has been hotly debated and disputed since it was proposed in 2013 (Green et al. 2013; Mackley and Capps 2017; Isidor, Julia et al. 2019; Koplin, Savulescu, and Vears 2020). While some – including the American College of Genetics and Genomics (ACMG) – consider testing for SF to be beneficial for improving patients’ health (Green et al. 2013; Kalia et al. 2017), others have questioned whether there is sufficient evidence that such variants will actually increase disease risk to warrant testing, which may lead to unnecessary investigations (Isidor et al. 2019).

All of these factors (and others) are likely to have implications for the pretest counseling interaction between patients and genetic health professionals (GHPs). One of the primary practice goals of genetic counseling involves obtaining the informed choice of clients (Resta et al. 2006), and research suggests it is both a key component of genetic counseling consultations and also a key concern of genetic counselors who are undertaking testing using genomic sequencing (Bernhardt et al. 2015; Sanderson et al. 2019). Indeed, genetic counselors have argued that obtaining informed consent for genomic sequencing is distinct from other genetic tests (Bernhardt et al. 2015) and that use of genomic sequencing challenges the “models under which informed consent is typically obtained,” which suggests that it might be important to have forms specifically for use in genomic sequencing (Tabor et al. 2011).

Much of the research conducted to date exploring informed consent practices in genomic sequencing has focused on analyzing and determining the types of information that should be listed in consent forms, rather than the processes by which GHPs obtain informed consent from patients (American College of Medical Genetics and Genomics 2013; Ayuso et al. 2013; Appelbaum et al. 2014; Henderson et al. 2014; Vears, Niemiec et al. 2018; Vears, Niemiec et al. 2018). This has included a recent study which used a randomized controlled trial to compare the consent encounters using a “standard” consent form for a genomic research study with an adapted consent form (Turbitt et al. 2018).

There has, however, been surprisingly little in the way of empirical research exploring genetic health professionals’ perspectives on the ways in which integrating genomic sequencing into clinical practice has created new challenges, or exacerbated existing ones, when obtaining informed consent from patients. One study used a best–worst scaling task to survey which aspects genetic counselors find most challenging in the consent process and how they prioritize elements of consent when presenting parents with options for their child to undergo exome sequencing (Gore et al. 2019). Several studies have also interviewed genetic counselors and/or research coordinators who work in either research or clinical settings about their experiences with the process of obtaining informed consent from patients (Bernhardt et al. 2015; Dheensa et al. 2018; Wynn et al. 2018; Sanderson et al. 2019). However, participant numbers are often small and many of these studies were conducted in the United States or the United Kingdom with limited exploration of experiences of genetic health professionals in other parts of the world. This may be important as guidelines from professional bodies, such as the ACMG (Kalia, Adelman et al. 2017) differ from those of Europe, Canada, and Australia, particularly in their stance on whether unsolicited findings should be reported and whether secondary findings should be actively sought (van El et al. 2013; Boycott, Hartley et al. 2015; National Pathology Accreditation Advisory Council 2017). In addition, the National Health Service in the United Kingdom is a unique healthcare system, meaning experiences of GHPs may be less relevant in other settings. As such, explorations targeting perspectives on non-US and non-UK based GHPs is warranted.

There is also limited exploration of GHPs experiences with informed consent in the clinical setting, as the existing studies mainly interview health professionals obtaining consent for research. Authors have proposed that, based on the translational nature of large-scale genomic research projects, and the significant overlap in clinical and research processes uncovered within some Clinical Research Evidence-Gathering Research (CSER) projects, there is little reason to draw a hard distinction between research and clinical care (Angrist and Jamal 2015; Berrios et al. 2018; Wolf et al. 2018). However, the primary goal of testing in the clinical setting is still to identify any potential underlying genetic contributions to the condition seen in the patient, whereas in research the goal is to generate knowledge. This difference is likely to impact the types of results that laboratories offer to return to patients and, as such, both the consent forms and the consent interactions would need to be adapted to be relevant to the clinical setting (Turbitt et al. 2018). For this reason, it is appropriate to explore the contexts separately – only then can the two be compared to determine how the differences between clinical and research settings need to be accommodated.

In order to contribute to the sparse literature on this topic, we present data from interviews with GHPs about the challenges they experienced when obtaining consent from their patients for genomic sequencing in the clinical setting.

## Materials and methods

We used qualitative methods to explore the experiences of genetic health professionals requesting diagnostic genomic sequencing for patients. Genetic health professionals were recruited using a purposive sampling strategy to identify both genetic counselors and clinical geneticists who request different types of genomic sequencing technologies for patients, including targeted large and small gene panels, exome sequencing, and genome sequencing, primarily in the clinical setting. Sampling aimed to recruit participants with considerable experience offering genomic sequencing to patients, with representation across different countries (within Europe) and States/Provinces (within Australia and Canada). As such, potential participants were identified using three strategies: 1) internet searches to identify genetic services - DV approached potential participants via email if their online profile suggested that they offer genomic sequencing to patients; 2) snowball sampling – participants were asked to nominate other potential participants at interview completion; 3) participants from a previous study of laboratory personnel (Vears, Sénécal et al. 2017) were asked to suggest GHPs that they knew were offering genomic sequencing to patients. As the consent process in clinical care and research are different (Bernhardt et al. 2015), we sought to recruit GHPs working within the clinical setting, although some were also involved in clinical research.

Interviews were semi-structured in nature and were conducted by one member of the research team, either in person or via telephone (DV). These interviews explored a range of topics related to GHPs’ requests for genomic sequencing, including the types of patients for whom they request testing, which types of tests they request, and which results they receive from laboratories. We also asked about their experiences returning results to patients, how reanalysis is initiated within their service, and what they found most challenging about requesting using genomic sequencing within their practice. The interview guide (available as a supplementary file) was developed based on gaps identified in the literature and also issues in practice highlighted by our previous interviews with laboratory scientists (Vears, Sénécal et al. 2017; Vears, Sénécal et al. 2017). Interviews were audio-recorded, transcribed, and analyzed using inductive content analysis; content categories were derived from the data, rather than pre-determined (Downe-Wamboldt 1992; Schamber 2000; Graneheim and Lundman 2004). Each transcript was coded into broad content categories. Sections of the data within the broad categories were compared and more specific subcategories were developed. All transcripts were coded by one researcher (DV) and a subset of the transcripts were coded by two other researchers to confirm the coding scheme.

Here we report one broad category that we identified from the data, namely the issues GHPs raised about obtaining informed consent from patients undergoing genomic sequencing that appeared to be either specific to, or exacerbated by, obtaining consent using genomic sequencing compared to standard genetic testing. Data reporting the GHPs’ experiences requesting genomic sequencing and receiving reports from laboratories (Vears, Senecal et al. 2020), and their experiences with initiating reanalysis of patient data (Vears, Senecal et al. 2020) have been reported elsewhere.

Verbal informed consent was obtained from all participants. This study was approved by the SMEC Review Board (Social and Societal Ethics Committee), KU Leuven and by the Research Ethics Board of the Faculty of Medicine, McGill University.

## Results

### Participant characteristics

Thirty interviews were conducted with 31 genetic health professionals (24 clinical geneticists and 7 genetic counselors), which included participants from 30 different institutions in Europe (15), Australia (10), and Canada (5). Participants had a mean of 9.5 years’ (1– 30 years’) experience in their current role and a mean of 14.3 years’ (3.5 − 38 years’) experience in the field of genetics. Of these participants, eight were involved in the analysis and interpretation of genomic sequencing data as part of their role and an additional nine assist with patient review at multidisciplinary team (MDT) or other types of meetings with their local laboratory. Fifteen of the GHPs worked solely with pediatric patients, 4 solely with adults, and 12 with both adults and children. Most (n = 29) participants requested varying combinations of exomes and panels (either virtual/bioinformatic or exon capture). Of the remaining two participants, one only requested and returned results from large panels while the other requested testing from a laboratory that used solely genomes.

### Challenges in obtaining informed consent in genomics

The GHPs described four main categories of challenges associated with use of genomic sequencing that impact on their ability to obtain informed consent from their patients: 1) the scale of the technology, 2) preparing patients for results, 3) engaging patients in the informed consent process, and 4) questioning the purpose of consent forms.

#### Scale of the technology

GHPs described how when using genomic sequencing, whether this involves exome, genome, or even large panel sequencing, the volume of data is much greater than they have often been exposed to previously. This was problematic for some participants, particularly those who are also involved in data analysis or interpretation as part of their role. They discussed how the increased volume of data increases the time required for interpretation of the variants to determine whether or not they are relevant to the clinical presentation of the patient. Even those GHPs who were not involved in the data analysis process often still needed to be able to “sift through” and interpret the information provided by the laboratory on the report, which could be quite labor-intensive depending on the level of detail provided and number of variants identified in the analysis.

As this clinician flagged, the entire process from the first interaction with the patient to result disclosure is more time-consuming since the introduction of genomic sequencing:

*What I am struggling with a lot with next generation sequencing, and I think everyone is, is that the time to consent, the preparation of gene lists or patient phenotypes in order to submit a test to the laboratory, and the liaison that’s required is much more time consuming. And then at the other end, giving results is slightly more time consuming as well… We’re much more commonly finding answers now which means that we have to go into a bit of detail and so I think on average, because we’re getting more results, on average the time for consultation is taking longer.*

*Participant 22, Clinical geneticist*

#### Preparing patients for results

Some GHPs mentioned that the additional time required during the informed consent process is partially due to the need to explain the types of results that patients might receive as an outcome of the test. It was highlighted that although there is a much greater chance of identifying a causative variant using genomic sequencing, there are still a significant proportion of patients for whom no cause will be identified. In line with this, participants described how an important component of the informed consent process is managing expectations for the patients. This included preparing patients for the potentially low likelihood that a genetic cause will be identified and also for the high chance that, even if they do identify a causative variant, this will not impact patient management. Participants explained that patients do not always seem to appreciate that even positive results may not be the answer that they were looking for.

*We do exome analysis on a lot of patients that we know very well that come very regularly to our clinic and they always explained to us the importance for them of having the diagnosis… And it’s very surprising when we give them back the results that a large majority of them are not so happy because they realize that having the results will not give them a solution for treating that child… Because, in a large majority, it’s ultra-rare disease where we really have not a lot of information. And in fact, we realize that the diagnosis was not an end for them. The diagnosis was really a wish, because they hoped for something else after the diagnosis.*

*Participant 8, Clinical Geneticist*

GHPs said that some of the main challenges they experience relate to the fact that use of genomic sequencing, particularly less targeted approaches, such as exome or genome sequencing, increases the chance of identifying two additional types of findings which were much less common using standard sequencing technologies. The first was that genome sequencing can identify VUS, i.e., where the current knowledge means that we are unable to classify whether or not the variant is relevant to the clinical presentation of the patient. GHPs discussed how the uncertainty associated with a VUS can be very challenging for clinicians to describe to patients, particularly in the pretest counseling phase. Yet, they felt it was important to prepare patients for VUS because, depending on the laboratory’s reporting policy, it is a relatively likely outcome of the test. Although some GHPs felt that patients had a relatively good understanding of what a VUS is, others felt that, overall, VUS are difficult for patients to fully grasp.

*I think VUS are really poorly understood. I think you can really try as hard as you like to explain it. You can be the best explainer in the world and it’s still lost on a lot of the general public, honestly.*

*Participant 15, Clinical Geneticist*

In addition, more open analysis of genomic sequencing data also carries with it an increased chance of identifying an unsolicited finding, i.e., where a genetic change is found that is unrelated to the clinical presentation of the patient yet shows that they are at increased risk of developing another genetic condition, such as hereditary breast and ovarian cancer. Again, as this is a potential outcome of the test, genetic health professionals generally felt that this likelihood should be discussed with patients prior to testing taking place. Although some of the GHPs felt the patients do understand the nature of unsolicited findings, others highlighted the difficulty of trying to explain the potential for unsolicited findings to patients during the informed consent procedure in enough detail that they can make an informed decision about what they want to receive. As this genetic counselor described, unsolicited findings can be particularly challenging to explain when the patient (or their family) is grappling with their diagnosis and may not have the capacity to take on health-related information extraneous to this.

*So, despite my best counseling skills and both myself and other clinicians talking about it, (the parents) were still very much on the page of that we’re looking for the cause of her epilepsy and that’s all that (the test) is going to look at… As much as we try to prepare our families and our patients for the possible outcomes, I don’t think that they really can appreciate that until they’re living that experience. So, you can sit there for an hour and talk to a family about “oh, there’s a possibility that, because we’re looking all the genes, we might find a mistake in a heart gene even though we’re looking at the cause of her epilepsy.” And people say, “oh yes, well that would be good to know” and “we’d want to know that because knowledge is power” and so on… I think still when it does happen, people are just not as prepared as they think they might be, if that makes sense. I think because they’re living the experience of some really bad conditions, I don’t think that they have capacity to think about anything else.*

*Participant 14, Genetic Counselor*

#### Engaging patients in the informed consent process

Several of the genetic health professionals worked in a setting where there was not a distinct boundary between clinical care and research for at least some of their practice. Participants practicing in this clinical-research space discussed their observation of a lack of willingness by patients/participants to engage with the informed consent process.

*A lot of the people that I’m speaking to are very eager for an answer so sometimes I have to slow them down to go through the consent form. Basically, they want to sign the paper, get the bloods, get the test started because they want to get it done quickly, basically, and they don’t understand why I’m making them go through all of this and why I keep talking to them! I should just be quiet and give them a pen.*

*Participant 24, Genetic Counselor*

GHPs described ways they attempt to manage this “balancing act” between providing sufficient information to facilitate informed choices without exceeding what is feasible within the clinical consultation. Despite their efforts, some still held concerns about how “informed” their patients/participants’ choices were regarding participation.

*I’m sure you’ve seen it 100 times. You give them the form, they look at it, they sign it, and they give it back to you… So, we summarize it. We try to make them feel comfortable about what we’re doing. It isn’t true informed consent I don’t think. And I think everyone agrees that it’s not with genomic testing.*

*Participant 15, Clinical Geneticist*

*There’s a need to find a nice balance between giving the patient informed choice but also being able to go through the consent process in a reasonable clinical amount of time.*

*Participant 26, Clinical Geneticist*

Some GHPs also questioned whether, given the complexity of genomics, some degree of paternalism is appropriate in order to assist patients in their decision-making.

*I don’t think it’s bad to be a little bit paternalistic, to help people make a choice in this, or to guide them or something. I think people need some guidance in this. It’s a dual feeling. But I think the general knowledge of genetics in the general population is really not that good so I think it would take a long time, like a complete consultation, to discuss the full scale of this decision with people to really get them to understand the full scale of their decision that it would be almost impossible to do that every time I think.*

*Participant 30, Clinical Geneticist*

#### Questioning the purpose of consent forms

Finally, some participants believed that the consent forms themselves may be more focused on protecting laboratories than informing patients.

*Yeah so, we have our own (consent forms) and it makes it easier because we know we’re covering everything that we need to cover. Because some of these consent forms from the labs are either very focused on disclaimers from the labs about all the things that they can’t see… It’s more to protect the lab than to inform the patient.*

*Participant 25, Clinical Geneticist*

*But yeah, having a consent (form) is just protecting us, no? It’s not protecting the patient… Of course, if you want to publish something, they want to see it. That’s the problem.*

*Participant 29, Clinical Geneticist*

## Discussion

Our findings show that genetic health professionals experience a range of challenges associated with the use of genomic sequencing in their patients and research participants that have an impact on the informed consent process. One major challenge discussed by our participants was the additional time pressure created by the use of genomic sequencing in practice. Participants mentioned tasks such as the preparation of gene lists (based on patient phenotypes or detailed phenotypic checklists) that are required for them to submit a test, liaising with laboratory staff, and sifting through the data on reports – all of which contributed significantly to their workload. This is supported by an Australian study, based on a census of 354 genetic counselors and clinical geneticists, which found that an additional 2.75 and 2.25 hours were added to GHPs’ workloads per patient for exome and genome sequencing respectively. Most of this additional time was spent on tasks relating to gene list development, prioritization, and curation, searching for evidence to support variant pathogenicity, and attending multidisciplinary team meetings (Nisselle et al. 2019). Interviews with 14 genetic health professionals in Australia and the UK also identified increased preparation time prior to returning results and additional investigations in order to confirm variants as factors specific to genomic sequencing that significantly impacted their practice (Dwarte et al. 2019). Although this in itself does not directly impact the informed consent process, it adds to the workload of the genetic health professionals overall, creating time pressure which may mean that GHPs feel that they have less time to spend with patients.

Another main challenge highlighted by our participants was the amount of information that needed to be covered in the pretest counseling/informed consent process to sufficiently prepare patients for the results that they might receive. This was because the nature of genomic sequencing (particularly exome and genome sequencing) increases the chance of identifying both VUS and UF compared to traditional, non-high throughput sequencing technologies. This accords with the findings of the best-worst scaling task conducted by Gore et al. who identified that managing expectations was one of the top challenges faced by the genetic counselors in their study (Gore et al. 2019). This finding also supports those of Bernhardt et al. who identified that their genetic counselor-participants found it challenging to educate their patients/participants about the full range of potential outcomes of genomic testing (Bernhardt et al. 2015). Similarly, genetic and non-genetic clinicians returning results from genomic sequencing as part of the NIH funded Clinical Sequencing Exploratory Research (CSER) Consortium projects discussed the need to spend more time preparing participants for the potential for uncertain results in the pretest counseling/informed consent process in genomic sequencing (Wynn et al. 2018). Two studies, one interviewing a range of medical specialists across four sites in the UK, and another interviewing genetic health professionals in both the UK and Australia, have highlighted the extended time needed to conduct the consent process for genomic sequencing as a prominent challenge (Dwarte et al. 2019; Sanderson et al. 2019).

Unlike the genetic counselors in the study by Bernhardt et al. (Bernhardt et al. 2015), the major challenges identified by our participants regarding the informed consent process did not include helping patients make decisions about which categories of secondary findings they wanted to receive. This is probably because, at the time of the interview, very few of the European and Australian laboratories from which the GHPs were requesting genomic sequencing for their patients were offering to actively search for SF. This is in contrast to laboratories in the United States where it appears that many (if not most) laboratories routinely offer to search for SF, although the nature of the SF offered and the ability for patients to choose which SF they desire varies between laboratories (O'Daniel et al. 2017). Despite the currently slow adoption of actively searching for SF by laboratories based outside of the US, recent studies in France and the UK that are doing so suggest that this is likely to become more common in the near future (Sanderson et al. 2019; Thauvin-Robinet et al. 2019). As such, assisting patients to determine which SF they desire during the consent process is likely to become more prevalent in practice, which may increase the time pressure felt by GHPs during the genetic counseling consultation. Furthermore, Turbitt et al. found that obtaining informed choice from a cohort of patients for secondary findings in the US may be more difficult to achieve than for the primary indication (Turbitt et al. 2018), suggesting that this may require more attention during the consent process than it is currently afforded.

Our participants discussed their difficulties engaging patients in the consent process, often in the context of patients that were also signing up to participate in clinical research studies in an attempt to identify the genetic cause of their, or their child’s, condition. Genetic counselors in Bernhardt’s study also described similar difficulties “maintaining participants’ attention” (Bernhardt et al. 2015) and focus groups with a range of healthcare professionals in the UK suggested that patients’ lack of understanding of genetic testing may be, in part, due to a lack of focus during the consent process (Samuel et al. 2017). This is likely to be partly due to both the keenness of the patients to find the cause for their condition, and also because the information that patients need to understand in order to make a truly informed decision is vast and complex due to the nature of the test, the range of potential outcomes, and the settings in which testing is taking place (Turbitt et al. 2018; Yu et al. 2019). Our GHPs discussed their attempts to manage this “balancing act” between the provision of “enough” information to allow patients/participants to make informed choices about testing, while not running over time or overloading the patient. They also discussed strategies, such as summarizing the information on the consent form and highlighting the key points, raised by others (Bernhardt et al. 2015). This ties into the comments raised by our participants who questioned the purpose of using consent forms to gain informed consent from patients for genomic sequencing, suggesting that they are more for legal protection than for the benefit of the patient. This accords with results of focus groups with health professionals in the UK, where some participants said they felt that consent forms gave them a sense of reassurance that they were protected against legal ramifications (Samuel et al. 2017). Are consent forms for genomic sequencing merely a way to protect laboratories from litigious patients? Kaye et al. stress the importance of consent forms for promoting the ethical principles of autonomy and respect for persons (Kaye et al. 2015). As such, we suggest that, while consent forms for genomic sequencing should be comprehensive, they should be viewed as a tool to support the informed consent process, facilitated by good genetic counseling, rather than as a stand-alone document.

Finally, the complexity of genomic information has led to postulations that some degree of paternalism in obtaining consent from patients was justified. At first glance, this appears to contrast with findings by Gore et al., who found that the genetic counselors in their study valued components of the informed consent process that encourage patient engagement and facilitate shared decision-making (Gore et al. 2019). This difference may reflect the fact that our sample was mainly comprised of clinical geneticists who have usually not received specialized training in genetic counseling. Nonetheless, our findings prompt consideration of the degree to which it is appropriate to guide patient/client decision-making. In the past, non-directiveness was viewed as a principle of genetic counseling practice (Biesecker 2001). However, this concept was removed from genetic practice guidelines in 2006 because it was felt to be incompatible with some areas of genetic counseling where directiveness *was* seen as appropriate, such as recommendations for screening in hereditary cancers (Resta 2006). Non-directiveness had also received considerable criticism over the years because it was often misconstrued as a practice of lumping patients with a lot of information and then leaving them to make decisions on their own and without support. Current guidelines propose that the primary goals of genetic counseling include “promoting understanding, facilitating decision-making, achieving client informed choice, reducing psychological distress, enhancing perceptions of personal control, and advancing adaptation to health-threatening information and experiences” (Biesecker, Peters, and Resta 2019). As such, there might be some scope for clinicians/counselors to provide guidance to patients/clients during the informed consent process. However, the aim of doing so would be to achieve the above goals, and the degree to which guidance was provided would need to be cognizant of this aim. With this in mind, rather than GHPs instructing patients which types of results they should opt to receive from genomic sequencing, this guidance would involve assisting patients to identify their own values and beliefs, tailoring the content of the discussion to provide information that addressed these, and assisting the patient to choose a course of action that accords with these values (Biesecker, Peters, and Resta 2019).

While the exploratory nature of our study limits its generalizability, it provides key insights into the types of challenges being experienced by GHPs when obtaining consent for genomic sequencing from patients. Although it is possible that some of the participants lacked experience obtaining consent from patients (and therefore the challenges they faced were not those of other GHPs), most of the participants had many years of experience in their roles – so it is unlikely that the challenges faced by our sample are due to their lack of skill. The experiences of our participants are not likely to be vastly different to those of GHPs overall.

As we have shown, there remains a tension between a) providing patients with highly specific and detailed information during the consent process, which risks leaving them overwhelmed by more information than they can take in, and b) providing patients with less information, which is easy to take in but is also less specific (and may therefore fail to provide the level of detail required to make an informed choice about whether to undertake the test or which results to receive). Although this tension is not new, as we have illustrated, it is exacerbated in genomics due to the complexity of the test, the sheer scale of the data, the range of possible outcomes of the test, and the requirement to manage patient expectations around these. Some authors have argued that the range of possible findings is so great that it is unrealistic to expect GHPs to secure *fully* informed consent from patients (in the sense of providing comprehensive information about possible findings and ensuring patients understand it) (Dondorp and De Wert 2013; Dive and Newson 2018). In our view, a more appropriate approach would be to focus on realizing, as far as possible, the underlying goal(s) that informed consent is meant to promote.

This raises the question of what the underlying goals of informed consent actually are. There may be several. One core goal is to promote participants’ autonomy (Savulescu and Momeyer 1997), which GHPs can achieve by providing participants with the information and support that will help participants make decisions that align with their own values. While the promotion of autonomy is widely considered central to informed consent, it is not necessarily the *only* goal of informed consent requirements. Other possible goals include the promotion of patients’ welfare interests and the promotion of public trust in medicine (Dickert et al. 2017).

There are important distinctions between each of these goals. Accordingly, they sometimes conflict. For example, if we understand the moral purpose of informed consent (mostly) in terms of promoting wellbeing, we might be comfortable with a greater degree of paternalism than if we understand the moral purpose of informed consent (mostly) in terms of promoting autonomy. Sometimes, however, they overlap. Crucially, the goals of promoting autonomy, wellbeing, and public trust in genomics are each consistent with providing patients with the kind of information they would find most helpful to their decision-making (and thereby promoting wellbeing, autonomy, and trust alike). Where the standard of *fully* informed consent might suggest one should supply some maximum amount of (potentially highly technical and complex) information, focusing on informed consent's underlying goals would instead suggest seeking the best possible balance between comprehensiveness, comprehensibility, and clarity.

Current consent processes may strike a less than ideal balance between comprehensiveness and comprehensibility. A study by Turbitt et al. has suggested that a shorter and lower density consent form developed for the research setting can be just as good at informing participants about study results as the standard consent form (although it showed low levels of informed decision-making regarding return of secondary findings) (Turbitt et al. 2018). Others have suggested novel approaches such as “layered” consent models, which provide essential information to all patients and additional information to patients who wish to learn more (Bunnik, Janssens, and Schermer 2013). One fruitful way to evaluate different consent forms and processes would be to consider how effectively they balance the goals that informed consent is meant to promote, such as beneficence and respect for autonomy, among others.

This approach would also be useful in the domain of genetic counseling, which has seen long-running debates about how strictly the norm of non-directiveness ought to be followed (Bartels et al. 1997; Weil et al. 2006; Bunnik, Janssens, and Schermer 2013; Jamal, Schupmann, and Berkman 2019). Here, too, it is useful to take a broader view of the varied goals that genetic counseling ought to promote in order to consider when non-directiveness is appropriate. We propose that non-directiveness has a legitimate role to play in genetic counseling insofar as it promotes some of these goals, such as respecting patients' autonomy. But this role needs to be balanced against other moral considerations, such as the effect on patients' welfare interests.

We think it would be valuable for future work to continue to explore what the underlying goals of informed consent are and how these goals can best be realized by GHPs and others. One core task here is to develop specific strategies for balancing comprehensiveness and comprehensibility. Another is to identify what kinds of information and interactions participants in existing trials find most useful. In addition, as these interviews highlighted concerns of GHPs about patients’ ability to provide truly informed consent, it would be worthwhile exploring the experiences of patients (and parents) who have received genomic sequencing with the informed consent process to see if these concerns are warranted.
